# The dopamine transporter gene *SLC6A3:* multidisease risks

**DOI:** 10.1038/s41380-021-01341-5

**Published:** 2021-10-14

**Authors:** Maarten E. A. Reith, Sandhya Kortagere, Corinde E. Wiers, Hui Sun, Manju A. Kurian, Aurelio Galli, Nora D. Volkow, Zhicheng Lin

**Affiliations:** 1 Department of Psychiatry, New York University School of Medicine, New York City, NY 10016 USA; 2 Department of Microbiology and Immunology, Drexel University College of Medicine, Philadelphia, PA 19129 USA; 3 Laboratory of Neuroimaging, National Institute on Alcohol Abuse and Alcoholism, Bethesda, MD 20817 USA; 4 Department of Psychiatry, Perelman School of Medicine, University of Pennsylvania, Philadelphia, PA 19104 USA; 5 Molecular Neurosciences, Developmental Neurosciences, Zayed Centre for Research into Rare Diseases in Children, UCL Great Ormond Street Institute of Child Health, and Department of Neurology, Great Ormond Street Hospital, London, WC1N 1EH UK; 6 Department of Surgery, University of Alabama at Birmingham, Birmingham, AL 35294 USA; 7 National Institute on Drug Abuse, Bethesda, MD 20817 USA; 8 Laboratory of Psychiatric Neurogenomics, McLean Hospital, and Department of Psychiatry, Harvard Medical School, Belmont, MA 02478 USA

**Keywords:** Genetics, Addiction

## Abstract

The human dopamine transporter gene *SLC6A3* has been consistently implicated in several neuropsychiatric diseases but the disease mechanism remains elusive. In this risk synthesis, we have concluded that *SLC6A3* represents an increasingly recognized risk with a growing number of familial mutants associated with neuropsychiatric and neurological disorders. At least five loci were related to common and severe diseases including alcohol use disorder (high activity variant), attention-deficit/hyperactivity disorder (low activity variant), autism (familial proteins with mutated networking) and movement disorders (both regulatory variants and familial mutations). Association signals depended on genetic markers used as well as ethnicity examined. Strong haplotype selection and gene-wide epistases support multimarker assessment of functional variations and phenotype associations. Inclusion of its promoter region’s functional markers such as DNPi (rs67175440) and 5’VNTR (rs70957367) may help delineate condensate-based risk action, testing a locus-pathway-phenotype hypothesis for one gene-multidisease etiology.

## Introduction

Dopamine (DA) plays a crucial role in multiple brain functions [1–4] and is also implicated in circadian rhythms and sleep [5, 6], inflammation [7, 8], heart failure [9, 10] and cancer [11–13]. Since the plasma membrane human DA transporter protein (hDAT) is one of the principal regulators of synaptic DA transmission [14], genetic variation in the coding gene, *SLC6A3* in the human chromosome 5 (chr5), may affect *SLC6A3* function, alter hDAT’s density, DA reuptake activity, and the dynamics of DA neurotransmission, contributing to pathophysiology in both central (CNS) and peripheral nervous systems.

Varying *SLC6A3* sequence has been correlated to many environment-sensitive psychiatric diseases such as substance use disorders (SUDs), major depressive disorder (MDD), attention deficit hyperactivity disorder (ADHD) and Parkinson’s disease (PD). Observed comorbidity presents tremendous clinical challenges [15]. In vivo activity of *Slc6a3* (low case for animal genes) is regulated by environmental risk factors including stimulant drugs [16–18], stressors [19, 20] and high-fat food [21] (increasing its activity), environmental enrichment (decreasing its activity) [22], as well as medications [23–25]. However, these regulations remain mechanistically unclear for humans with the related diseases.

Understanding *SLC6A3*’s functional variants is required to delineate individual variation in DA-related pathophysiology and response to the environment [26, 27]. To enhance our understanding, we performed a risk synthesis. Risk synthesis is not risk analysis, review or meta-analysis alone. Instead, it utilizes available information from various approaches, published and/or newly collected, to clarify disease mechanisms in humans. At least three infectious disease studies have partly applied this format of study at behavioral and/or molecular levels [28–30]. This risk synthesis study utilized functional evidence from eight *SLC6A3*-focused approaches, including familial hDAT mutants (mostly review) with their molecular modeling (new data), case-control associations (review), recombination hotspots of *SLC6A3* (new data), hDAT imaging vs. genotype correlational analyses (new data), postmortem mRNA density (new data), transcriptional assays in vitro (review), phylogenomics & intragenic epistases (new data), and intrinsically disordered regions (IDRs) of transcription factors (TFs) analysis (new data). The Methods section below will describe the collection of the indicated new data.

## Methods

**Fig. 1:**
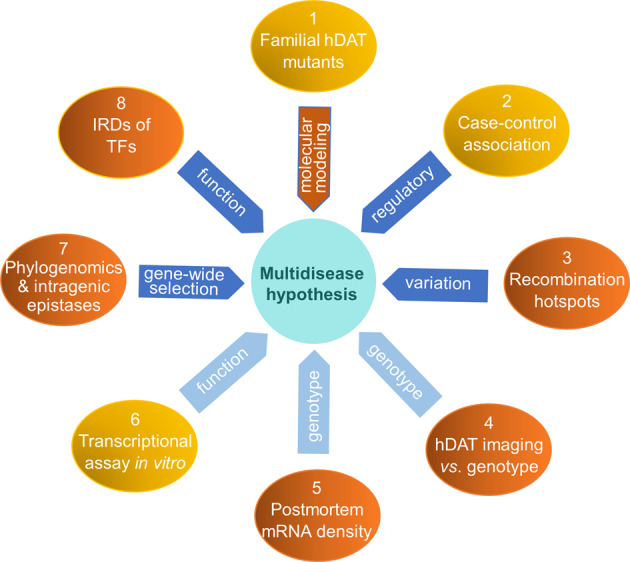
Study design. Eight channel-synthesis of risk knowledge for functionally polymorphic and vulnerable *SLC6A3*, allowing proposing of a multidisease hypothesis. Mustard oval, reviewed data; orange (1/molecular modeling, 3, 4, 5, 7, 8), new data whose collections are described in the same order in Methods section; blue arrow, mechanism; light blue arrow, functional approach; IDRs of TFs, intrinsically disordered regions of transcription factors.

By its methodological nature the risk synthesis relies on both data already available in the literature and newly collected information via secondary analysis of previously collected samples or data (for study design, see Fig. 1). This section describes the methods used for such secondary analyses and new data collections. Synthesis of all eight-approaches’ information allows proposing a hypothesis for *SLC6A3*’s multidisease mechanism.

### Molecular modeling of familial hDAT mutants

**Fig. 2:**
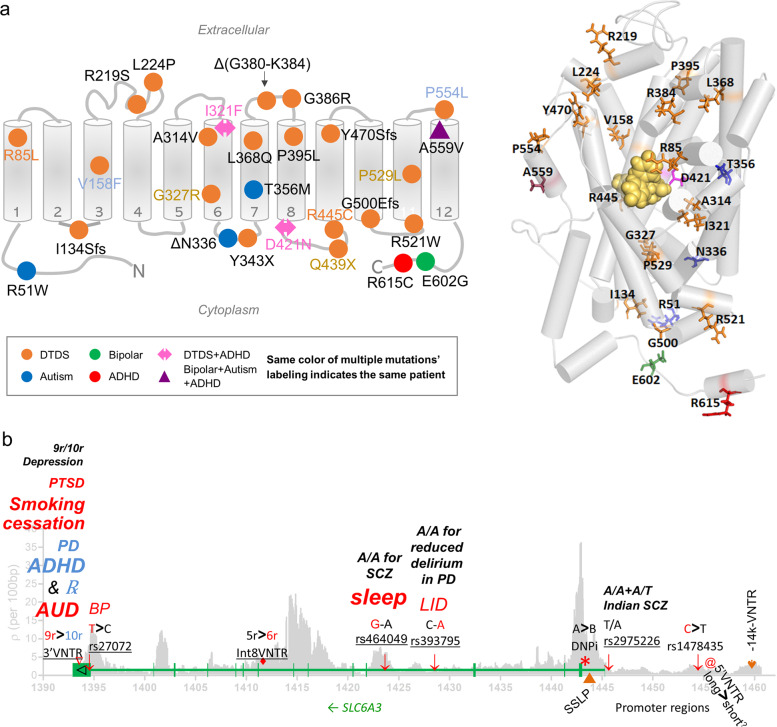
Functional syntheses for association signals. **a** Molecular modeling of familial hDAT mutants for structural interpretation. (left) Two-dimensional distribution of mutations. Each gray cylinder represents a transmembrane (TM) domain; upside, synaptic space; downside, cytosol of DA neuron. (right) Three-dimensional structural model of mutated residues with TM helices represented by gray cylinders. Mutated residues are represented as licorice sticks and colored using the same convention as in (left panel): DTDS in orange, autism in blue, ADHD in red, BP in green and the combined mutants for autism, BP and ADHD in magenta and labeled. The substrate/inhibitor site that includes both S1 and S2 sites is represented by the docked inhibitor represented in space filling model and colored yellow (these represent new information - see Supplementary text for Fig. 2a in the Supplementary Information). **b** Associated (underlined), functional (>) or other complex (orange symbol) markers in regulated *SLC6A3*. *SLC6A3* has 15 exons (14 introns), with the start codon in Exon 2 and the stop codon in Exon 15 so that its 1863 bp-coding region ends with the first 23 bp of the 2 kb long Exon 15 (based on GenBank Accession # NM_001044). Indicated are functional markers in red and other unique complex markers in orange: 3’VNTR and rs27072 in 3’UTR, Int8VNTR, rs64049 and rs393795 in Intron 4, DNPi and SSLP (▲) in Intron 1, rs2975226 in core promoter, rs1478435, 5’VNTR (@) and -14k-VNTR (♥) in distal promoter regions. SNPs are indicated with ↓. Green, *SLC6A3* gene structure (vertical bars are 15 exons in the opposite strand of the chromosome); >, variant activity greater than; -, unknown activity for two alleles; /, a genotype associated; italics, associated diseases or response to medication (℞) (BP bipolar, METH methamphetamine use disorder, LID levodopa-induced dyskinesia, SCZ schizophrenia); bold, meta-analysis result and font size indicates significance or sample size used where same color matches risk allele with phenotype;?, only postmortem mRNA correlational data; gray: recombination rate (new data) obtained from combining all 25 1KGP populations, below with the chr5 position in kb. Paucity, lack of association efforts in promoter regions (no familial mutants are included). Only association studies with meta-analysis statistical significance or reproduceable results are summarized here.

Drosophila melanogaster DAT structure (pdb code 4XP4 [31]) was used to model the wildtype (WT) hDAT and each mutation using homology modeling techniques with Modeler (ver 9.23), followed by structural refining using Molecular Operating Environment, as previously reported [32], and modeling results are detailed by Fig. 2a and associated supplementary text information.

### Recombination hotspots

Polymorphism information in 26 populations (2268 individuals informative for this gene) of the 1000 Genomes Project (1KGP) [33] was used to reveal recombination hotspots in *SLC6A3* by using the published FastEPRR protocol [34]. The results are integrated in Fig. 2b.

### hDAT imaging *vs*. genotype correlational analysis consisted of the following four steps

1. Positron emission tomography (PET)
  Measures of hDAT availability in the human brain had been collected using PET and [^11^C]cocaine, a radioligand to measure hDAT, and retrieved from the imaging dataset of the BNL Brain Imaging Center. All PET scans were performed on a Siemens, HR + scanner in 3D mode via procedures as reported [35] along with the analytical approach to quantify the availability, which was estimated as *B*_max_/*K*_D_ [36, 37]. For this study we only used data obtained in 62 healthy male controls (average age at 35.1 years with a standard deviation of 6.7; *N* = 27 African Americans, 28 Caucasians, 3 Hispanics, and 4 multiracial).
2. Genotyping of DNPi and 5’VNTR
  By using REDTaq ReadyMix™ PCR Reaction Mix from Sigma, DNPi was amplified by using primers 5’-gaatacagatgaacagtcatgaagac-3’ and 5’-ctcatgggcacactgggagttgagg-3’, and 40 cycles of 94° C-30 sec, 58° C-30 sec, and 72° C-45 sec, both using 72° C-10 min as the final PCR extension. DNPi product was subject to allelic *Bse*RI (New England Biolabs, Ipswich, MA, USA) digestion at 37^o^ C overnight. 5’VNTR was amplified in reported primers [38] through 40 cycles of 94° C, 30 sec, 60° C 30 sec and 72° C 45 sec. All DNA fragments were resolved by electrophoresis in 1.1% agarose gels.
3. Haplotyping of DNPi and 5’VNTR was performed by using SHEsis [39].
4. Statistical analyses were done using SPSS Statistics Subscription version 26 (IBM, Armonk, New York, USA). Multivariate analyses were performed with genotype/haplotype as the between-group variable and hDAT availability in striatal subregions as dependent variables. For 5’VNTR, repeats were combined as a quantitative variable. Age and gender were added as covariates. Post hoc univariate tests and *t* tests were performed to investigate directions of effects. *P* < 0.05 were considered statistically significant.

**Fig. 3:**
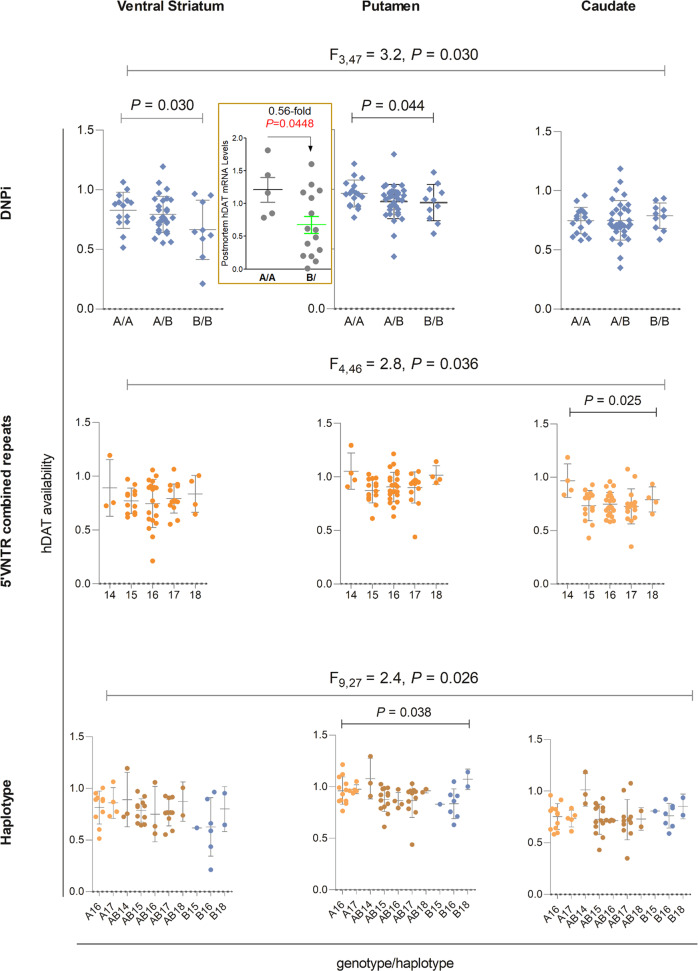
Genotype-correlation of hDAT availability (Bmax/KD in *Y* axis) in healthy ventral striatum (left), putamen (center) or caudate (right). Insert, postmortem hDAT mRNA levels in isolated nigral DA neurons from twenty independent healthy subjects; upper, DNPi; middle, 5’VNTR; bottom, haplotype of DNPi and 5’VNTR; multivariate results for three brain regions are given in *F* values and only significant *P* values are shown (*N* = 62). Between-genotype differences were not statistically significant after Bonferroni corrections. After excluding women (*N* = 3), the effects remained for men. These are all new data.

Postmortem mRNA density of hDAT in laser-capture microdissection (LCM)-isolated postmortem DA neurons from 20 healthy subjects had been previously estimated [40] and its correlation with DNPi genotype is presented in Insert of Fig. 3 upper panels.

### Phylogenomics & intragenic epistasis analyses

**Fig. 4:**
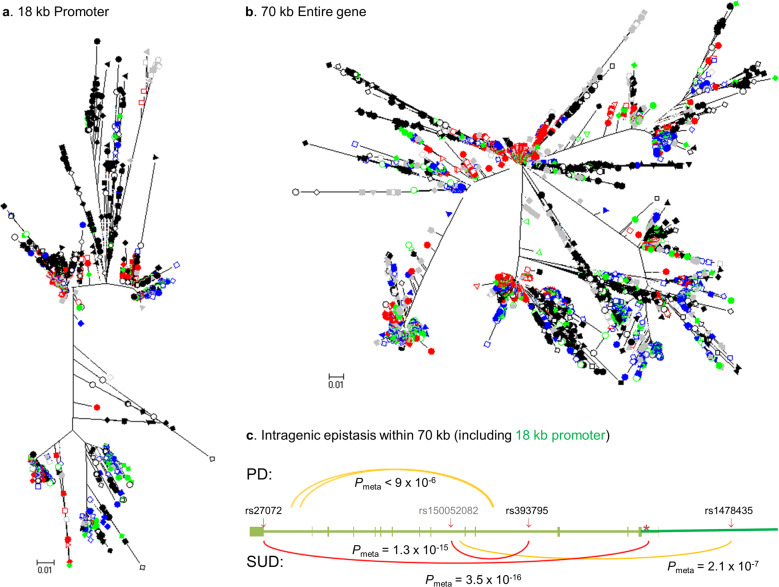
Genetic evidence for functional variants throughout *SLC6A3*, based on haplotype selection of 18 kb promoter (a) or 70 kb whole gene (b) and on case-control intragenic epistases for PD and SUDs both in Caucasians (c). In first two panels of phylogenic trees, labeling of the 26 1KGP populations are explained as follows. East Asians all in red: closed circle for Chinese Han Beijing (CHB); open square, Japanese (JPT); closed square, Chinese Han South (CHS); closed diamond, Chinese Dai in Xishuangbanna (CDX); open triangle, Kinh in Chi Minh City, Vietnam (KHV). European ancestry all in blue: closed circle, Utah residents with Northern and Western ancestry (CEU), open diamond, Toscani in Italia (TSI); closed triangle, Finnish in Finland (FIN), closed square, British in England and Scotland (GBR); open square, Iberian population in Spain (IBS). African ancestry all in black: closed circle, Yoruba in Ibadan, Nigeria (YRI); closed diamond, Luhya in Webuye, Kenya (LWK); closed triangle, Gambian in Western divisions of the Gambia (GWD); inverse closed triangle, Mende in Sierra Leone (MSL); closed square, Esan in Nigeria (ESN); open circle, Americans of African Ancestry in SW USA (ASW); open diamond, African Caribbeans in Barbados (ACB). Admixed Americans all in green: closed circle, Mexican ancestry from Los Angles USA (MXL); closed diamond, Puerto Ricans in Puerto Rico (PUR); open circle, Colombians in Medellin of Colombia (CLM); open triangle, Peruvians in Lima of Peru (PEL). South Asian all in gray: closed circle, Gujarati Indian in Huston of Texas USA (GIH); closed diamond, Punjabi in Lahore of Pakistan (PJL); closed square, Bengali of Bangladesh (BEB); open square, Sri Lankan Tamil from the United Kingdom (STU); and closed triangle, Indian Telugu from the United Kingdom (ITU). In panel (**c**), 6572 unrelated subjects in three cohorts were used for PD (above the gene schematic related to Fig. 2b) and 5843 in another three cohorts, for SUDs (below the gene schematic). Shown are intragenic epistases selected largely for those involving Fig. 2b-mentioned markers where yellow indicates suggestive significance and red, for statistical significance. Unlabeled SNPs are rs11564770 (at 1398806) and rs11564772 (at 1398007) in Intron 14 or last intron, and rs250686 (at 1425159) in Intron 4 for PD; rs11564757 (160 bp downstream of DNPi indicated by asterisk) in Intron 1 and rs2975292 (at 1419932) in Intron 6 for SUDs. rs150052082, in gray, was a new SNP which interacted with multiple loci in SUDs, see Supplementary Table 1 for details. These are all new data.

Polymorphism information in the 26 1KGP populations was used to generate phylogenic trees via ClustalX and TreeView [41, 42]. Intragenic epistases (case-control epistatic associations) were evaluated by meta-analyses of logistic regression results, as previously described [43] except only focusing on Caucasians in this study (the African American cohort was excluded). *P*_meta_ < 0.05 from Bonferroni-based multiple-testing was considered as statistically significant. Data are described in Fig. 4 and in Supplementary Table 1.

IDRs of TFs prediction was carried out using the MobiDB software [44] and the data are presented in Supplementary Table 2 in the Supplementary Information.

## Results and discussion

### Genetic associations

To reveal genetic risks in *SLC6A3*, two types of association studies have been carried out, familial mutations and case-control associations.

#### Familial mutations

During the last decade, 26 hDAT mutants have been identified mainly in individuals with dopamine transporter deficiency syndrome (DTDS) [45] (Table 1) [46–64].

**Table 1:** Familial hDAT mutants identified in patients with DTDS, ADHD, autism spectrum and bipolar disorders (ordered by ascending mutant DA uptake activity in % of WT).

Clinical case # ^k^ (numbering in ref)	Mutant(s)	DA uptake	Functional alteration in transporter properties	Phenotype	Age at	Lifespan	Gender	Ethnicity	Mutations	*SLC6A3*	Reference
	in clinical case	% of WT^a^		in patient	onset	(years) (date last reported)			in cDNA^m^	location in Exon	
1 (5)	V158F	0	Mature hDAT ^b^↓,						610G>T	4	[46]
			surface hDAT ↓	DTDS class., inf. PD Dystonia ^c^							
					0.33	8.9 (†)	F	Mixed European			
	P554L	0	Mature hDAT↓↓,						1799C>T	13	
			surface hDAT ↓								
2 (10)	L224P	0	Mature hDAT↓↓,	DTDS class., inf. PD Dystonia	0.58	12.75 (2021, still alive though significantly disabled)	F	Mixed European	809T>C	5	[46]
			surface hDAT ↓								
3 (1,2: two first cousins)	L368Q	0	Mature hDAT↓↓,	DTDS class., inf. PD Dystonia	0.06, 0.25	15, 13 (2020, both still alive)	M, F	Pakistani	1241T>A	8	[46, 47]
			surface hDAT ↓								
4 (6)	Unknown non-hDAT product	(0)^d^							(425-5_425-2delCACAinAACG)	(Intron 2 splice mutation)	[48]
				DTDS class., inf. PD Dystonia	0.25	13 (2020, still alive)	M	Italian			
	G386R	0	Mature hDAT↓↓						1294G>A	8	
5 (3)	P395L	0	Mature hDAT↓↓,	DTDS class., inf. PD Dystonia	0.42	20 (2020, still alive)	F	Mixed European descent	1322C>T	9	[46, 47]
			surface hDAT ↓								
6 (7)	Y470Sfs	0	Mature hDAT↓↓,	DTDS class., inf. PD Dystonia	0.25	4 (2014)	M	Mixed European	1546_1547insAG	3	[48]
			surface hDAT ↓								
7 (7)	p.I134SfsX5	(0)^d^	ND^d^	DTDS class., inf. PD Dystonia				Mixed European	537delG	3	[46]
					0.12	14.2 (†)	F				
8 (8)	p.G500EfsX13	(0)^d^	ND^d^	DTDS class., inf. PD Dystonia	0.4	11 (2020, still alive)	F	Mixed European	1637_1905del	12,13	[46]
9 (6)	Unknown non-hDAT product	(0)^d^	ND^d^	DTDS class., inf. PD Dystonia	0.21	15 (†)	M	Turkish	(1169+1G>A)	(Intron 7 splice mutation)	[46]
10 (4)	Unknown non-hDAT product	(0)^d^	ND^d^	DTDS class., inf. PD Dystonia	0.33	16.2 (†)	F	Mixed European	(1294+5delG)	(Intron 8 splice mutation)	[43]
11 (4,5: two sisters)	Unknown non-hDAT product	(0)^d^	ND^d^	DTDS class., inf. PD Dystonia	0.25, 0.17	35 (2014), 10 (†)	F, F	Mixed European	1407+1G>A	(Intron 9 splice mutation)	[48]
12	R219S	0	In silico predicted to have	DTDS class., inf. PD Dystonia	at birth	27 (2020)	M	Italian	793C>A	5	[49]
		(in silico predicted)	damaged function								
13	p.G380_K384 delinsE	0	In silico predicted change in splicing/gene expression and possibly damaging impact on transport function by deletion in EL4	DTDS class., inf. PD Dystonia	0.17	≥5.5 (2019)	F	Iraninan	1277_1288del	8: in-frame deletion of 12 bp	[50]
		(in silico predicted)									
14	Y343X	(0) ^d^	ND ^d^	DTDS class., inf. PD Dystonia	0.33	>3 (2020)	M	Iranian	1167C>G	7	[51]
15	Δ N336	0	AMPH-DA efflux ^e^ ↓; stabilization of a more open intracellular gate; conformational flexibility ↓; N336 flies display hyperlocomotion, grooming and anxiety ↑, and social interaction ↓	Autism spectrum disorder	<16	≥16 (2019)	M	European	1146CAAG>G	7	[52]
16 (9)	G327R	0	Surface hDAT ↓						1117G>A	7	[46]
	Q439X	0		DTDS class., inf. PD Dystonia with clinical response to cocareldopa	0.25	16.5 (2020, still alive)	F	Mixed European descent	1453C>T	10	
	P529L	6.2	Mature hDAT↓↓,						1724C>T	12	
			surface hDAT ↓								
	G327R-439X with P529l										
		1.7									
17 (8)	R85L	0.5	Mature hDAT ↓,						392G>T	2	[48, 53]
			surface hDAT ↓;								
				DTDS class., inf. PD Dystonia	0.75	9 (2020, still alive)	F	Mixed Ashkenazi, Jew/ Iranian,/ Yemeni/ Turkish			
	R445C	5.6	Surface hDAT ↓; loss of transport due to block of N-terminus release						1471C>T	10	
	R85L + R445C	1.8									
18 (1,2,3: three brothers)	A314V	8.8	Mature hDAT↓,	DTDS atypical, juvenile PD Dystonia					1079C>T	7	[48]
			surface hDAT ↓		11, 11, 11	16, 26, 28 (2014)	M, M, M	Pakistani			
19 (11)	R521W	26.9	Mature hDAT↓,	DTDS class., inf. PD Dystonia with clinical response to cocareldopa					1699C>T	12	[46]
			surface hDAT ↓, syntaxin interaction ↓		0.42	15 (2020, still alive)	F	Mixed European			
20	I312F	56 (V_max_)	Outward bias ^f^	DTDS atypical, adult early-onset PD Rigidity; ADHD^h^	28	≥ 40 (2014)	M	Mixed European	1072A>T	7	54, 55
	D421N	10	Inward bias; loss of Na^+^ and Cl^-^ binding; a constitutive effluxer^g^						1399G>A	9	
	I312F + D421N	30									
21	T356M	42 (V_max_), 34 (V_max_)	Outward bias; AMPH-DA efflux ↓ but a constitutive effluxer; T356M^++^ mice show hyperlocom-otion, repetitive rearing, and loss of preference for social novelty	Autism spectrum disorder	<6	≥6 (2013)	M	Caucasian	1205C>T	8	[55–57]
22	R615C	62 (V_max_), 102 (V_max_)	Accelerated rate of endocytosis and recycling; alteration in hDAT microdomain localization	ADHD						15	[55, 58]
					<13	≥13 (2012)	M	Caucasian	1981C>T		
23 ^l^ (two unrelated individuals and twenty-one neuropsychiatric patients)	K619N	69 (V_max_)	AMPH-DA efflux ↓; mature hDAT ↓; surface hDAT ↓; K619N flies display hyperlocomotion; mutation is in PDZ-binding domain	Autism spectrum disorder; DTDS atypical, adult early-onset PD, personality disorder with schizophrenia and depression; association with bipolar disorder	?, ~ 39, ?	?, 53 (2020), ?	?, M, ?	?, Caucasian, ?	1995	15	[59]
24 ^l^(single individual, two siblings, two probands for a total of 5 individuals)	A559V	100 (V_max_), 104 (V_max_)	AMPH-DA efflux ↓ but a constitutive effluxer	bipolar disorder; ADHD; autism spectrum disorder	≤18, ?, ?, ?, ?	≥18, ≥6, >6, ≥18, >18	F, M M, M M	German, Caucasian, Caucasian, ?, ?	1814C>T	13	[55, 60–62]
25 (two siblings)	R51W	94 (V_max_)	AMPH-DA efflux ↓; syntaxin interaction ↓	Autism spectrum disorder	?, ?	?, ?	M, F	?, ?	289C>T	2	[63]
26	E602G	0	Only properties studied and found to be normal:	Bipolar						14	[55, 62, 64]
		99 (V_max_) ^i^	K_m_, V_max_,		≤18	≥18	F	German	1943A>G		
			binding psychostimulants,								
			binding Na^+^ and Cl^- j^								

Note: This table presents reviewed data except some of the lifespan information updated in 2020 or 2021.

^a^Uptake is expressed as % Vmax of wild-type (WT) if uptake was appreciable enough to estimate K_m_ and V_max_ values (generally K_m_ was unaltered); otherwise data are expressed as % uptake of WT measured with low nanomolar [DA].

^b^Mature hDAT: fully glycosylated protein with a molecular weight ~80 kDa.

^c^Classical (Class.) Dopamine Transporter Deficiency Syndrome (DTDS); marked by infantile (inf.) onset and parkinsonism (PD) dystonia as opposed to a typical DTDS that starts later in childhood with milder parkinsonism dystonia symptoms.

^d^Because there was no hDAT product, it could not be heterologously expressed and studied (ND); by definition, uptake activity was 0.

^e^Amphetamine (AMPH)-induced DA efflux.

^f^hDAT takes on different conformations during transport and mutants can have a bias for more outward- or inward-facing conformations.

^g^Anomalous DA efflux, or constitutive DA efflux.

^h^Attention Deficit Hyperactivity Disorder (ADHD).

^i^The two discrepant uptake values of 0 and 99 were obtained with different cell systems, HEK-293 (stable transfection) and COS-7 cells (transient), respectively; the value of 99% for this mutant was obtained along with those for 5 other ADHD/autism mutants examined under the same conditions and is the one accepted here for comparison purposes. The reported value of 0 was accompanied by pictures of cellular distribution showing the absence of hDAT at the cell surface, in a single stably transfected cell line.

^j^Other properties such as AMPH-DA efflux have not been studied.

^k^Unless indicated otherwise, one case is one person.

^l^Clinical data are grouped in same order separated by semicolon.

^m^Based on NM_001044 updated on 12-OCT-2020, with 138 bases as 5'UTR.

The first causal relation between functional deficiency of hDAT and a clinically relevant disorder was reported by us in 2009 [47] with family members carrying an homozygous mutation (Leu368Gln or Pro395Leu) affected with dopamine transporter deficiency syndrome (DTDS) whereas family members that were wild-type or heterozygous carriers did not show the clinical syndrome of DTDS. This appropriate segregation of mutation with disease status was observed for all of the subsequent DTDS cases studied by us, indicating causality [45] (Table 1 cases 1–11, 16–19) [46–64]. Typical DTDS patients display dramatic infantile phenotypes with progressively worsening dystonia and parkinsonism and are at risk of premature death in childhood or adolescence. Upon arranging all cases according to the severity of functional DA uptake, it can be seen that the first 19 mutants in the Table lost 100–70% reuptake activity, characteristic of DTDS except case#15 (ΔN336) with autism (see Fig. 2a legend). Some patients display atypical later onset parkinsonism (case# 18 and see Fig. 2a), with one patient additionally afflicted with ADHD (case# 20) [54].

Six mutations were implicated in mental illnesses only: Arg51Trp [63], Thr356Met [57], Ala559Val [60] and ΔN336 [52] in autism, Arg615Cys in ADHD [58], and Glu602Gly in bipolar disorder (BP) [62]. Thr356Met drove persistent reverse transport of DA (efflux) [57]; ∆N336 displayed impaired DA transport, reduced AMPH-induced DA efflux, diminished AMPH-induced currents, resulting in autism-related behaviors in Drosophila. Arg615Cys might increase membrane mobility [65]. Although there is no evidence that these mutations cause the observed mental illnesses, the rarity of the former mutations and their coincidence with a psychiatric disorder does suggest their role as a risk factor.

These mutations are located throughout the protein structure (Fig. 2a left panel), with the DTDS mutations mostly in the translocation path for reuptake activity, ion binding and/or surface localization (Fig. 2a right panel), explaining their impaired reuptake. The mutations associated with psychiatric conditions affect DA efflux and involve interactions with other proteins such as Gβγ subunits of the G-protein for Glu602Gly in BP and Arg615Cys in ADHD or syntaxin for Arg51Trp in autism [66, 67], affecting hDAT networking (see Supplementary Text for Fig. 2a). However, it remains unknown whether some of the phenotypes are also attributable, in part, to associated synonymous variations.

#### Case-control associations

Classic markers used were two variable number of tandem repeats (VNTRs), one in the 3’ untranslated region (3’UTR) commonly known as 3’VNTR of 40 bp (rs28363170), and another in Intron 8 or Int8VNTR of 30 bp (rs3836790). 3’VNTR has two common alleles, 9- and 10-repeat (9r,10r) and Int8VNTR also has two common alleles, 5- and 6-repeat (5r,6r). Different populations may have additional alleles in either case. Moreover, several single nucleotide polymorphisms (SNPs) in various regions have also been used as association markers. Considered here are only reproduced and statistically significant findings, as summarized in Fig. 2b. SNP-based genome wide association studies (GWAS) have not implicated *SLC6A3* in the neuropsychiatric diseases [68–70]. This was likely due to ethnicity or the fact that GWAS were based on SNPs and the associations with ADHD and alcohol use disorder (AUD), for example, are on 5’VNTR, not SNPs, given the high recombination rates between 5’VNTR and SNPs in other regions of this gene (see Fig. 2b).

#### A. Etiology

Most studies focused on diseases related to DA transmission in the CNS, based on a priori knowledge.

Substance use disorders (SUDs) AUD and tobacco use disorder (TUD) are two leading causes of chronic diseases so they became a main focus of *SLC6A3* association studies. In 2016, the Li Lab published two meta-analyses. One was on AUD in 5846 participants, concluding that 3’VNTR9r, the shorter allele, was the risk variant [71], which has been further supported by more recent findings [72, 73]. The other was on smoking cessation in 5480 participants, concluding that 9r was a promoting variant for cessation (pooled OR = 1.17) [74], which has been further supported by recent studies in 819 Chinese and 1230 Russian subjects [75, 76]. Therefore, both in AUD and TUD the 9r variant is a risk allele; a remaining question is whether this variant has a role in relapse vulnerability.

##### ADHD

A meta-analysis of 59 studies on 3’VNTR among a total of 31,457 children and adolescents with ADHD indicated that 10r is the risk allele [77]. The association was significant in Caucasians and in Europeans, but not in Asians.

##### Other complex psychiatric diseases

Studies employing meta-analyses have implicated this gene in posttraumatic stress disorder (PTSD) (3’VNTR9r OR = 1.62) [78], MDD (pooled OR = 1.26) [79, 80], schizophrenia (SCZ) (pooled OR = 3.2) [81], sleep duration (rs464049 G allele, beta [standard error, SE] = − 0.96 [0.18] minutes/allele; *P* = 5.71 × 10^−10^ as a genome-wide significance) [82] and PD [83]. An early Dutch study examined 16 SNPs plus 3’VNTR in 720 patients and uncovered selective rs393795 A/A protection against delirium in PD [84]. The study’s meta-analysis of three SNPs in 1641 patients indicated consistently and selectively that rs393795 A/A reduces delirium in PD (OR = 0.37) [85]. This gene apparently contributes to PD in multiple ways. In addition, rs27072, a SNP in 3’UTR, was implicated in BP [86]. Even though psychiatric diseases are multifactorial, all these studies point to an important risk coming from the 9- and 10-repeat alleles of the 3’VNTR of *SLC6A3*.

#### B. Response to medications

Several studies reported genetic effects of *SLC6A3* on response to medications. A meta-analysis of 36 studies with a total of 3647 youth for genetic modulations of methylphenidate’s efficacy in ADHD treatment found that in 16 studies, the ADHD-risk variant 3’VNTR10r was associated with reduced efficacy (OR = 0.74) [87]. Clearly, taking into account such individual genetic variability may improve the success of childhood-ADHD treatment with methylphenidate (Ritalin).

Another pharmacogenetic involvement was found for levodopa in PD patients. Two recent reviews agreed that *SLC6A3* contributed to the response to levodopa in various cohorts [88, 89]. For example, results in 352 levodopa-treated Israeli PD patients indicated that the C allele of rs393795 extended significantly the time to levodopa-treated dyskinesia (LID) onset, time ratio = 4.96 (95% CI 2.3–10.9, *P* = 4.1 × 10^−5^) [90]. Findings from recent studies further supported the association of this *SLC6A3* variant with LID [90, 91]. Moreover, *SLC6A3* may interact with other DA concentration-regulating genes (*SLC18A2* and *COMT*) in treatment-related complications [92]. Although the risk of LID is a consequence of a number of intrinsic (patient-related) and extrinsic (medication-related) factors, these studies point to an important contribution to the risk by genetic effects of *SLC6A3*.

### Common regulatory variants

To clarify disease mechanisms, the first step is to investigate which variants display altered activity or respond to a regulator. More than 10 polymorphisms have been identified as risk variants in association studies but only three of them have been assessed for in vitro *cis*-acting or transcriptional regulatory function.

#### Three known polymorphisms

##### 3’VNTR cis-activity: 9r is higher than 10r

3’VNTR, the first *SLC6A3* marker used, has been the most studied, followed by Int8VNTR. Two groups reported consistently that 3’VNTR9r enabled higher in vitro CMV promoter activity than 10r [93, 94], which is supported by studies in HEK293 cells that showed 9r to display higher promoter activity than the 10r allele; however, both showed lower activity than the control (without the VNTR), suggesting the 40bp-repeat is *cis*-inhibitory [95].

Imaging studies in humans can clarify the relationship between 3’VNTR and hDAT abundance. Many disease conditions, including SUDs, can affect gene activity and disrupt this relationship so studies must first evaluate the relationship in healthy individuals. A meta-analysis of imaging findings from 12 studies in a total of 511 individuals reported that the 9r variant correlated with increased hDAT protein level in healthy individuals by either PET or SPECT but this relationship was attenuated in affected individuals (patients with ADHD, AUD, PD and SCZ) [96]. The latter suggests that there is an additional, unknown factor that comes into play after the disease has developed, i.e. the risk from *SLC6A3* is important in developing the disease.

##### Int8VNTR cis-activity: 5r is higher than 6r

Studies of in vitro heterogeneous promoter activity in SH-SY5, reported that the shorter allele 5r displayed higher hDAT activity activity than the longer allele 6r [97]. This data was consistent with a finding from a reporter analysis in the mouse substantia nigra-derived dopaminergic cell line SN4741 [98].

We have clarified in humans a relationship between Int8VNTR and hDAT abundance by PET imaging of 95 healthy subjects with the consistent result that 5r was associated with higher hDAT availability than 6r [99].

The above studies utilized heterogenous promoters, not the human promoter, for the allelic functions. We have looked at the allelic effects by combining the shorter alleles or combining the longer alleles on the entire human *SLC6A3* promoter in 18 kb of two haplotypes (A and B); we found that the human promoter carrying the 9r + 5r together displayed higher promoter activity than one carrying the 10r + 6r together, especially in the 18 kb B haplotype and in SN4741 [100].

PET studies of healthy individuals confirmed independently that 10r + 6r-carriers had the lowest hDAT availability [99], establishing that the shorter alleles confer higher promoter activity than the longer alleles under basal conditions. Given the findings that these VNTRs both are inhibitory [100], it is plausible that the shorter alleles confer higher promoter activity by carrying less inhibitory activity with the smaller number of repeats.

##### rs27072 cis-activity in 3’UTR: T is higher than C

Rs27072 is a common variant with an average minor allele frequency (MAF) of around 20% by 1KGP. In a BP association study Pinsonneault et al. [86] assessed postmortem mRNA levels, used an in vitro promoter reporting assay, and measured DA uptake activity; the results indicated that the minor allele T is a risk variant, displaying an enhanced hDAT activity.

#### Three new markers around the promoter

We recently reported several novel and common polymorphisms around the promoter regions [38, 101] and three of them already have functional implications.

##### DNPiA-B (rs67175440) cis-activity in humans: A is higher than B

DNPi (dinucleotide polymorphism in Intron 1) has two alleles A and B (A: AG, B: GA as two adjacent bases of rs2975223T/C and rs2937640C/T) [38], a very common variant with an average MAF of 39% (12–50%, 1KGP). A long non-coding RNA (lncRNA, termed ^AZI2^3’UTR) DNPiB (rs2975223T) allele-dependently inhibited the *SLC6A3* promoter activity in vitro related to SUDs [102], suggesting DNPiB is a new functionally inhibitory variant.

PET imaging re-analysis showed an overall multivariate effect of DNPiB rs2975223 on hDAT availability (F_3,47_ = 3.2, *P* = 0.030). Post-hoc comparisons indicated elevated hDAT availability in the DNPiB rs2975223 A/A group compared to B/B in putamen (*P* = 0.044) and ventral striatum (*P* = 0.030). hDAT availability was also higher in ventral striatum for A/B than B/B at trend level (*P* = 0.056). These protein-based imaging findings were confirmed by mRNA results in single postmortem nigral DA neurons (Fig. 3 upper panels).

##### 5’VNTR (rs70957367) cis-activity: possibly more repeat, more activity

5’VNTR is located 11 kb upstream of Exon 1 and has 60 bp for 6–9 repeats [38]. Postmortem mRNA data has suggested it to be a functionally related marker, with a combined repeat number of two alleles from 15 to 18 positively correlated with the mRNA levels in controls, but not in cocaine abusers [38]. This finding suggests that 5’VNTR may function as an enhancer.

PET imaging re-analysis showed an overall multivariate effect of 5’VNTR on hDAT availability (F_4,46_ = 2.8, *P* = 0.036). Univariate analyses showed this effect was present for caudate (F_4,52_ = 3.1, *P* = 0.025), mainly due to higher levels in 14-repeats-carriers (Fig. 3 middle panels). The 15–18 repeats showed a consistent tendency of more repeats, more activity and in fact, post hoc *t* tests showed a significant group difference between 15 and 18 in putamen (*P* = 0.042). The main group differences were largely due to the combined 14-carriers.

Haplotype analysis of DNP1 and 5’VNTR still suggested a genetic effect on hDAT availability (F_9,27_ = 2.4, *P* = 0.026), especially in putamen (*P* = 0.038) (Fig. 3 bottom panels). These statistical significances disappeared after Bonferroni correction.

##### rs1478435 C-T cis-activity: C is higher than T

Located at 9057 bp upstream of Exon 1, rs1478435 is a common variant with MAF of 26% (T) per 1KGP. The C allele carries an enhancing activity on the *SLC6A3* promoter, related to SCZ [40]. By contrast, the T allele was associated with reduced gene activity based on reduced mRNA levels in postmortem nigral DA neurons from healthy individuals and reduced transcriptional activity based on 2.5 kb *SLC6A3* promoter reporting assays in two cellular models. We have not looked at it in human subjects yet.

These new and verified 5’ markers may allow us a superior ability to detect association signals. In addition, a simple sequence length polymorphism (SSLP) in Intron 1 and a -14kb-VNTR represent two novel common markers [101], worthy of functional investigation. Collectively, state of the art risk information including allelic activity under basal conditions is now aligned with the association signals in Fig. 2b.

### High vs low activity variant as a risk

Several studies [103, 104], including ours [40, 105, 106], have shown increased *SLC6A3* activity associated with brain disorders such as AUD, PTSD, BP and SCZ except ADHD or smoking cessation. In fact, chronic DA depletion is noticed already as an underlying factor for SUDs [107–110], which can be a result of increased *SLC6A3* expression or a reduced dopaminergic tone. Consistently, known environmental risks such as the most established risks stressors and nicotine could increase *Slc6a3* activity in vivo [19, 111–113].

Like tobacco smoking, cannabis use in adolescents may become a gateway by increasing the vulnerability for SUDs including AUD and OUD in later life [114–116]. Such gateway effects may occur through adaptation processes in DA neurons [117]. Interestingly, cannabinoid receptor type 2 (CB2) is expressed in DA neurons and modulates cannabis’ addictive effects [118]. Deletion of CB2 from DA neurons reduced alcohol preference [106] and *Slc6a3* activity [119] in mice, suggesting that cannabis’ effects may be mediated through the activation of *SLC6A3* but also that activation of *SLC6A3* may be part of the adaptation processes. This information again supports the view that increased *SLC6A3* activity could be a risk for SUDs.

A recent study reported a decreased hDAT level associated with depression [120]. However DAT knockdown reduced anxiety and depression-related behaviors in mice [121, 122], supporting a view that reduced hDAT levels protect against depressive behaviors. Such contradicting results suggest the possibility that the clinically observed decrease could be a result rather than a cause of the disease, representing another example for disease effects on gene activity.

Decreased gene activity can be a risk too, for diseases like Caucasian ADHD. These findings are not surprising considering ethnic medicine and polygenetic disorders. It is noted that a simplistic mode of action such as up- or down-regulation is unable to explain various association findings, warranting investigation of the dynamics of hDAT regulatory networks.

### Choice of markers

Given the fact that there are >2500 *SLC6A3* SNPs per 1KGP, design of a case-control association study needs to evaluate markers for three reasons.

First, many markers have no functional implication yet, including whether any risk pathways can regulate them in an allele-specific manner. If they are not the underlying variants, the association signals could be linkage disequilibrium (LD)-guided results. *SLC6A3* has many recombination hotspots, which occur among the common markers and separate the downstream VNTRs from the promoter regions (Fig. 2b) [101]. For a solid association signal, it is important to use the underlying functional marker or a marker close to or of strong LD with the underlying locus.

Second, given multiple functional variants, it comes down to whether all need to be typed especially when some are enhancing activities and others are inhibitory. This is because there could be intragenic antagonisms among these loci [123–125] so that use of one or few of them could be a biased study design. Regulation has only two end directions, up and down, regardless of the number of functional loci present but the key is its dynamics, when and what regulate the gene via these loci, partly defining the multidisease mechanism. Therefore, multimarker association analyses may enable gene-wide delineation of disease and comorbidity mechanisms.

The third reason is a technical aspect since some complicated markers are difficult to work with and impossible to type with a high throughput manner. *SLC6A3* has such complicated markers, which include the SSLP at +1531 in Intron 1 (nine known alleles) and the variable but complicated sequence arrangement −14kb-VNTR (four known common alleles) [101]. These difficult markers might be among the main functional variants contributing to phenotypes.

### Beyond one gene-one phenotype: the importance of regulatory contributions

Almost all the diseases mentioned above are polygenic phenotypes. Among the familial mutations, DTDS clusters carry severely impaired DA reuptake function; others carry 34% or more impaired function in cases of mental illnesses, likely involving other mechanisms such as efflux or co-existence with other genetic variations. Direct and indirect hDAT networks might be operating for the diverse, “*V*_max_-noncompliant” familial phenotypes.

On the other hand, pathway and cell subtype could mediate its regulatory genetics. Noticeably, 3’VNTR is related to seven phenotypes (Fig. 2b), with three possible explanations. First, a pathway targeting this locus is hub-connected with others playing critical roles among the seven phenotypes. Consistently, genetic pathways are increasingly recognized in etiology of diseases [126, 127]. Second, 3’VNTR plays different roles in different cell-types (e.g., caudate vs other striatal regions) [99] since DA neurons are heterogeneous and regulate different elements of brain function [128–130]. Third, some signals such for PTSD and depression arise from LD with their underlying loci. 3’VNTR is the most used marker and accounted for most signals here.

Overall, *SLC6A3*’s multiphenotypic findings can be explained not only by conditional hDAT activity but also by coordination with other genes and the impact of environment and epigenomics during certain sensitive periods in development [131, 132]. In other words, mutations in different parts of *SLC6A3* may affect multidimensional dynamics of the protein’s activity; or, a phenotype may stand out only when a *SLC6A3* functional variant co-exists functionally with other particular variation in the subject’s genome through pathway action. Those explanations warrant future study using a systems approach for mechanistic elucidation.

### Gene-wide functionality

We have shown that two different haplotypes of the 18 kb *SLC6A3* promoter may carry different gene functions and display different responses to the same drug regulators [100]. Based on the critical role of DA transmission, DA transporter haplotype and functional diversity could guide natural selection of genetic variants [133]. We thus examined the 18 kb promoter regions vs the entire 70 kb gene, for directed haplotype diversity among 26 1KGP populations.

The DNA sequence-based relatedness revealed >15 clades for *SLC6A3*, comparing to one major and one minor for the 18 kb promoter (Fig. 4a vs b). A key feature was that the haplotype diversity was mostly independent of ethnicity but few of the selections were indeed ethnic oriented, consistent with the ADHD findings. For example, Southern Asians or Africans each had at least two distinct selections. By contrast, Europeans and Admixed Americans lacked such selections. Since this approach may reveal functional variation [134], the result suggests that *SLC6A3* carry great functional diversity. Given the realization that associations can be ethnicity dependent for common diseases including depressive disorder and PD among others [83, 135–138], meta-analyses need to consider ethnic stratification for mechanistic clarification [138–140].

Consistent with the gene-wide selection results, significant epistatic associations with PD vs SUDs pointed to disease-oriented genetic coordination among different functional variants located from the 3’ to the 5’ ends (Fig. 4c). Together, these data suggest that functional variations are located in both promoter and non-promoter regions, which supports the association signals for diseases in Fig. 2b (throughout the gene) and for protein expression in Fig. 3 (promoter regions).

### Gaps in knowledge

Many pieces are missing in terms of genetic contributions considering pros and cons of LD-based and synthetic association [141], coordination with other genes [102, 142], concurrent epigenetics, cell type-dependence and perhaps polymorphic RNA properties, which have not been examined for this gene. Among them, three key gaps emerge given the current status of our understanding.

**Fig. 5:**
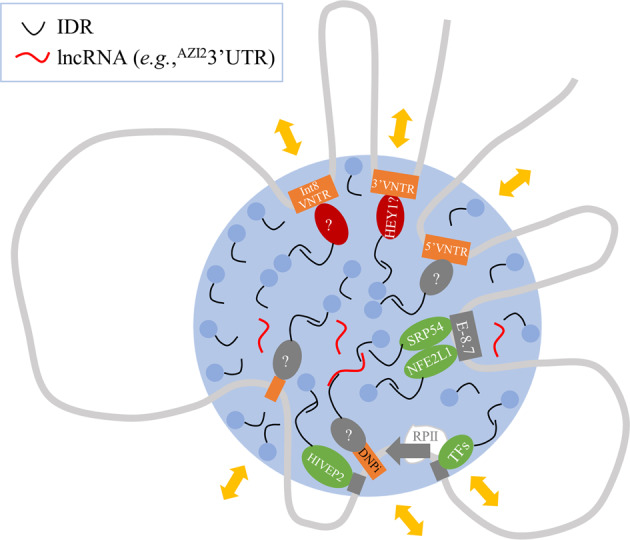
Multidisease hypothesis for variant- and pathway-related condensate in *SLC6A3* transcription. Gray curve, chromosome DNA harboring *SLC6A3*; orange rectangle, polymorphic sites (unlabeled for Intron 4); gray rectangle, *cis*-acting element; red oval, repressor; green oval, activator; gray oval, unknown TF as indicated by “?”; light blue dot, other condensation proteins such as Mediator; large blue circle, dynamic condensate; gray arrow, transcription start with RNA polymerase II (RPII) complex; TFs, other known transcription factors such as NURR1, GMEB1, PITX3, FOXA2, LMX1A and SP1/3. Yellow double-arrows, different disease-related and TF-mediated pathways. HEY1 has not been shown with allelic binding yet; majority of the proteins each have multiple IDRs so that this schematic has neither molecular nor dimensional accuracy.

Gap one is molecular engagement: multiple *cis*-acting loci need to be typed at the same time, that is, a multimarker approach considering the extensive genetic recombination (Fig. 2b). This way one could identify a locus-selective association and sort out phenotype-specific pathways. Recent developments in transcription mechanisms uncover the formation of liquid-phased condensate for gene transcription and this condensate may contain various proteins with IDRs and RNAs [143, 144]. Multivalent and weak interactions among the IDRs and RNA contribute to the phase formation. Consistently, known TFs of *SLC6A3* may contain IDRs (Supplementary Table 2), along with lncRNA [102], and are well positioned to contribute to such a condensate. This *SLC6A3* condensate, which engages both enhancers (such as E-8.7) and silencers (such as 3’ and Intron 8 VNTRs), may enable an initial understanding of a multisite mechanism (Fig. 5).

Gap two is causality. Most clinical investigations generate correlational information. To clarify the genetic contribution, allelic engineering studies in rodents or higher species are needed to examine the behavioral consequences. This approach is now possible with the new genomic editing technologies [145, 146].

Gap three is environmental genetics, an important role in the systems etiology. To fill this gap requires the use of genetically engineered animal models to evaluate the effects of relevant environmental factors.

### Conclusion and hypothesis

**Fig. 6:**
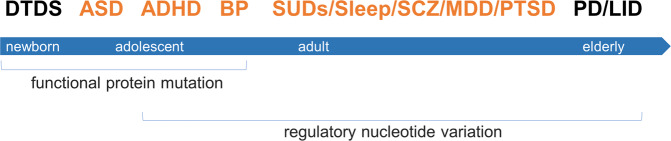
*SLC6A3* genetics x age-dependent expression of phenotypes. Horizontal blue bar, lifespan from newborn at left to elderly at right; associated phenotypes are indicated on the top where red, for psychiatric disorders and black, for movement disorders at the two vulnerable ends of lifespan; associated genetic effects are indicated below the bar; x, interaction.

*SLC6A3* contributes to a spectrum of central nervous diseases and comorbidity in two main ways (Fig. 6). Nonsynonymous mutations affect hDAT’s properties; synonymous variations each confer unique spatiotemporal features of the transcription, responses to internal or environmental factors or post-transcriptional RNA properties.

How does *SLC6A3* exert its risks? More information is missing than has been accumulated about this question, reminiscent of the parable “blind men and an elephant”. An emerging hypothesis is pathway-differential condensate (Fig. 5). By this hypothesis, several pathways may regulate *SLC6A3* condensate via TFs partly at polymorphic loci and those same pathways also intersect with other genes so that each pathway connects a set of different genes related with other diseases for shared genetic risks [147]. To test this locus-pathway-phenotype (LPP) hypothesis, we postulate that it requires consideration of multiple functional markers in the same association or functional study and stratification of ethnicities and genders [43].

## Supplementary information

Supplementary information

## Data Availability

Data collected or analyzed by this study are included in this published article (and its Supplementary Files).
