# Supplementary material for: The dopamine transporter gene SLC6A3: multidisease risks
Source: Mol Psychiatry. 2021 Oct 14;27(2):1031–46. doi: 10.1038/s41380-021-01341-5 (PMC9008071; doi:10.1038/s41380-021-01341-5)
Supplement: Supplementary file 1 — Supplementary information [file 41380_2021_1341_MOESM1_ESM.pdf]

Supplementary Information

Item	Page
Supplementary text for Figure 2a. Familial hDAT mutants: molecular modeling	2
1. Structural analysis	2
2. Regulatory mechanisms beyond hDAT	3
Supplementary Table 1. Intragenic epistases	4
Supplementary Table 2. IDRs of TFs	7

Supplementary text for Figure 2a:

1. Structural analysis

The mutations that have been identified in patients causing neurological or psychiatric conditions are mostly single point mutations except for a single residue or short truncation that affect both the structure and function of hDAT either by directly interfering with the substrate translocation pathway or allosterically by promoting conformational changes that renders the transporter less or non-functional. One of the common observations across all many of the mutants that code for DTDS is the reduced expression of mature or surface hDAT suggesting a role in the kinetics of the DA neurotransmission. Hence it is not surprising that most of the patients that have one or more of these mutations end up in developing Parkinsonian disease in later stages of their life. The mutants that code for psychiatric conditions affect DA efflux or psychostimulant binding or carry mutations in portions of the structure that interact with other non-hDAT proteins. In this study, we have made a concerted effort to map these mutations to hDAT structure and understand the likely implications on functions. However, it must be noted that these mutations are mapped to the modeled structure of hDAT using the dDAT or bacterial leucine transporter structures and hence have limitations to our complete understanding of the structure-function relationships in hDAT.

Additional mutations that code for either neurological or neuropsychiatric conditions are that could have a structural and/or functional effect are described below: Residue V158 in TM3 is proximal to the key residue Y156 which has been shown to be involved in both substrate binding as well as in maintaining the open outward conformation of the transporter (PMID: 17978168). V158F causes significant disruption to this site with steric clashes with residues C463 and W162. Similarly, introducing a proline (L224P) or removing it (P395L) produces structural changes that lead to rearrangement of loops leading to loss of coordination of Zn2+ binding (PMID: 10601246). Mutations of R85L and D421N in binding pockets affect substrate and Na+ binding. Mutating a glycine residue to arginine in G386R mutant in EL4, leads to steric clash with L440 and likely interactions with the polar lipid headgroup on the membrane. Mutation of residue P395 to a leucine which is located at the cusp of EL4 and TM8 induces a conformational change in the first half of EL4 loop and also upper half of TM8 since prolines are known to promote a kink as an imino acid. The effect of the conformational change on EL4 is reflected in the loss of coordination of E396 on Zn2+ binding (PMID: 10601246). The effect of this conformational change is observed TM8 given the prominent role played by Proline residues in conformation of a helix. Further, the mutant P395L poses a steric clash with F412 which is also proximal to the substrate binding pocket. For the EL2 residue R219, previous studies have shown that the corresponding residue in rat DAT R218 has a salt bridge interaction with D174 in a model based on the bacterial Leucine transporter structure (PMID: 24269640). However, in our model based on the dDAT, we do not observe this salt bridge interaction with R219 but do observe hydrogen bonded interactions with the neighboring E215 and H225 sidechains. Both these hydrogen bonded interactions are lost with the R219S mutation. The role of EL2 in DATs and other monoamine transporters has been shown to play a structural role and indirectly influence the transporter function (PMIDs: 23525969, 24269640).

Mutations R85L and R445C are situated diagonally opposite ends of the translocation pathway on TM1 and TM9 respectively (Figure 2a) at a distance of 33.6 A. In the dDAT based model of hDAT, R85 makes a salt bridge interaction with D385 which has been shown to be a conserved structural element among many monoamine transporters (PMID: 25339174) that contributes to the dynamics of the transporter during substrate binding and translocation. R445 makes a similar salt bridge interaction with E448 side chain at the entrance of TM9. Mutations R85L and R445C not only disrupt these salt bridge interactions but also indirectly affect the outer open conformation of hDAT. We have recently demonstrated R85 also forms part of the allosteric site (PMID: 31184115) that can regulate the substrate binding pocket through a network of hydrogen bonded interactions. Based on these interactions, we believe R85L or R445C mutation can directly impair substrate binding and transporter function. More specifically, R445 acts as a component of a latch in its formation with a salt bridge with E428 which helps enforce the inward closed state of the transporter; when this salt bridge is broken (as in the R445C variant), R445 acts as a trigger releasing the N-terminus allowing the inward open state (PMID: 29258773). In another recent study of ours (PMID: 34002696), the R445C mutation is shown to further disrupt the intracellular gating network endowing the mutant with channel-like properties that serve to mediate uncoupled ion fluxes.

Residues I312, A314 and D421 are proximal to the substrate binding region and mutations to these residues have a direct effect on substrate binding. For example, D421 is involved in the coordination of the Na+ ion binding which is required for substrate binding to the S1 site in the outward open conformation of hDAT. Mutation D421N has leads to loss of Na+ ion coordination causing a domino effect on the substrate binding and the stability of the outward open conformation.

Mutant A559V is localized on TM12 and is in proximity to the region that interacts with several adapter proteins. A study by Carneiro et al. (PMID: 12177201) showed that a region formed by residues 561-590 that encompasses TM12 and parts of the C-terminal region co-localizes and interacts with an adapter protein Hic-5 at the presynaptic terminal. The interaction of Hic-5 through its LIM domains has been demonstrated to be responsible for a reduction in hDAT uptake activity through a mechanism involving a decrease in the cell-surface levels of hDAT.

The single amino acid truncation of ΔN336 was modeled and structural superimposition of the mutant with WT hDAT showed that the mutant lies at third intracellular loop (IL3). In the WT, N336 forms hydrogen bond interactions with the side chain atoms of E122 at the end of TM2 and main chain atom of S333 thus forming a structural constraint between TM2 and IL3. The loss of N336 leads to removal of this structural constraint causes a movement of TM2 and IL3 away from each other and likely affecting the stabilization of the open conformation. In addition, N336 forms part of the YXXφ intracellular motif (with X being any amino acid and in this case, N336) that has been shown to be involved in transitioning of the transporter into different conformational states during the translocation cycle and the mutation of Y335A led to a transporter conformation that was stimulated by Zn2+ binding rather than inhibition (PMID:11818545). We hypothesize that the effect of deletion of N336 and the loss of the structural constraint between TM2 and IL3 will lead changes in these conformational states during translocation cycle.

2. Regulatory mechanisms beyond hDAT

Malfunction of hDAT, due to familial gene mutations, is encountered in patients with different types of diseases, including DTDS, autism, ADHD, and bipolar disorder (Table 1). When rank-ordering the mutants in Table 1 according to DA uptake activity, one quickly sees that DTDS clusters among mutants with only 0 to 30% of normal DA uptake activity (Table 1). Thus, most of the DTDS-related mutants are either dead transporters or have lost most of the reuptake activity, displaying typical infantile-onset of PD; A314V with 9% residual DA uptake is associated with atypical DTDS with juvenile onset of PD symptoms whereas the case that combines two mutations (I312F and D421N) has 30% of normal DA uptake and displays atypical DTDS with adult-early-onset PD along with ADHD. Two mutants (P529L and R521W) with 6% and 27%, respectively, residual DA uptake activity, occur in cases of typical DTDS that responded to cocareldopa treatment. Of note, the reduced interaction of R521W with syntaxin may have rescued some DA uptake function from the deleterious effect of the mutation (see Cervinski et al, 2010, PMID: 20643191). Importantly, all mutants listed in Table 1 other than the DTDS cases (with Δ N336 as the exception, see below), display residual DA uptake activity of 34% or more and have been observed in cases of autism spectrum disorder, ADHD, or bipolar disorder. Clearly mechanisms other than severely impaired DA uptake are at work in these latter disorders. If one looks at the cases with ADHD and/or autism together, there are seven mutants with this disease spectrum; three of those (D421N, T356M, A559V) have anomalous DA efflux, or constitutive DA efflux (ADE), but four other mutants (Δ N336, I312F, R615C, and K619N) do not. Please note that ADE is reduced by ritalin, a treatment for ADHD. It may be important to observe that any autism mutant has the property of reduced amphetamine-induced DA efflux (AMPH-DA efflux in Table 1, seen in Δ N336, T356M, K619N, A559V, and R51W). We do not understand fully the relevance of reverse DA transport (as revealed with amphetamine) but its reduction in autism could be important as it is the common property of hDAT in the autism mutants. Within the cluster of DTDS mutants with severely impaired DA uptake in Table 1, Δ N336 stands out as a mutant without DA uptake capability but at the same time, instead of generating DTDS, displaying autism spectrum disorder. Clearly, one gene, in this case *SLC6A3* with a lack-of-function mutation, does not necessarily lead to one disease as the phenotype. In the case of DTDS, it has been speculated previously by Blackstone (2009, PMID: 19504720) that lack of DA uptake ultimately leads to depleted storage vesicles so that neuronal impulse flow does not produce sufficient DA, resulting in PD symptoms. It is possible that this could be overcome by enhanced DA synthesis as observed in fruit flies carrying ΔN336 (Campbell et al., 2019, PMID: 30755521). Perhaps in the ΔN336 patient there is enhanced synthesis, just by being at the higher end of a natural range in synthesis activity, or there could be a function-enhancing mutation in DOPA decarboxylase or tyrosine hydroxylase, with either case leading to a sufficient restoration of the dopamine content of storage vesicles overcoming the zero DA uptake effect in producing PD. Finally, regarding the E602G mutant, it is unknown what functional properties of hDAT could be altered in relation to the observed bipolar disorder. Table 1 lists the only hDAT properties that were measured for E602G and found to be normal; unfortunately, no information has been reported on its AMPH-DA efflux capability or its potential ADE property, in order to see whether there is an overlap with the A559V mutant, the only other case with bipolar symptoms.

Supplementary Table 1. Top intragenic epistases of *SLC6A3* for PD & SUDs in Caucasians.

Disease	SNP1	Position 1*	SNP2	Position 2*	P_{meta}
**PD:**					
	rs11564770	1398806	rs250686	1425159	1.61E-06
	rs11564772	1398007	rs250686	1425159	8.85E-06
**SUDs:**					
	rs150052082	1419007	rs462053	1431554	1.09E-16
	rs150052082	1419007	rs461753	1431214	3.29E-16
	**rs27072**	1394522	rs11564757	1443762	3.50E-16
	rs150052082	1419007	rs458609	1434306	4.52E-16
	rs4975636	1388625	rs140029686	1390581	5.35E-16
	rs150052082	1419007	rs428280	1436476	5.77E-16
	rs150052082	1419007	rs638964	1433867	6.68E-16
	rs150052082	1419007	rs638577	1433831	7.04E-16
	rs150052082	1419007	rs487781	1433931	7.04E-16
	rs150052082	1419007	rs457702	1434430	7.31E-16
	rs150052082	1419007	rs465989	1432881	1.01E-15
	rs150052082	1419007	rs460007	1431853	1.06E-15
	rs150052082	1419007	**rs393795**	1428514	1.33E-15
	rs150052082	1419007	rs427284	1429187	1.47E-15
	rs150052082	1419007	rs460700	1429969	1.48E-15
	rs150052082	1419007	rs456082	1430515	1.58E-15
	rs150052082	1419007	rs250682	1427803	1.58E-15
	rs150052082	1419007	rs461677	1431992	1.62E-15
	rs150052082	1419007	rs459141	1432042	1.62E-15
	rs150052082	1419007	rs460000	1432825	1.62E-15
	rs150052082	1419007	rs464061	1430244	1.63E-15
	rs150052082	1419007	rs410209	1430775	1.63E-15
	rs150052082	1419007	rs458860	1430933	1.63E-15
	rs150052082	1419007	rs57106193	1431123	1.63E-15
	rs150052082	1419007	rs463379	1431164	1.63E-15
	rs150052082	1419007	rs458632	1431302	1.63E-15
	rs150052082	1419007	rs460934	1431306	1.63E-15
	rs150052082	1419007	rs465130	1432876	1.75E-15
	rs2975292	1419932	rs7737692	1461167	7.61E-15
	rs150052082	1419007	rs456774	1432202	9.15E-15
	rs73028257	1399091	rs12654851	1454004	1.79E-14
	rs150052082	1419007	rs10063564	1438030	1.55E-13
	rs150052082	1419007	rs10068876	1438042	1.55E-13
	rs2975292	1419932	rs748209	1457554	1.34E-12
	rs27072	1394522	rs6350	1443199	1.51E-12
	rs2042449	1416646	rs150052082	1419007	1.82E-12
	rs150052082	1419007	rs57212133	1425166	2.02E-12
	rs150052082	1419007	rs10053602	1428135	2.08E-12
	rs150052082	1419007	rs9312866	1436102	2.11E-12

rs150052082	1419007	rs10051340	1426166	2.34E-12
rs150052082	1419007	rs62331115	1433272	2.35E-12
rs150052082	1419007	rs2173947	1426959	2.38E-12
rs2975292	1419932	rs2937650	1458018	2.51E-12
rs150052082	1419007	rs61696543	1425313	2.51E-12
rs150052082	1419007	rs10475006	1425235	2.59E-12
rs150052082	1419007	rs10475005	1425049	2.69E-12
rs150052082	1419007	rs28382247	1420346	2.78E-12
rs150052082	1419007	rs28382246	1420382	2.78E-12
rs10040882	1418558	rs150052082	1419007	2.93E-12
rs12332091	1418745	rs150052082	1419007	2.93E-12
rs150052082	1419007	rs409588	1430834	4.25E-12
rs150052082	1419007	rs62331112	1424364	6.44E-12
rs2975292	1419932	rs905201	1457986	3.66E-11
rs2975292	1419932	rs4738	1461568	5.19E-11
rs28363174	1393038	rs409588	1430834	8.77E-11
rs40184	1395077	rs11564757	1443762	1.08E-10
rs1048953	1438174	rs28742608	1440934	5.47E-10
rs12654851	1454004	rs7737692	1461167	8.68E-09
rs28382245	1420476	rs11564751	1447223	3.44E-08
rs11564758	1420588	rs11564751	1447223	3.44E-08
rs28382245	1420476	rs6413429	1447027	5.18E-08
rs11564758	1420588	rs6413429	1447027	5.18E-08
rs4975646	1433401	rs6350	1443199	1.66E-07
rs461753	1431214	rs403636	1438354	1.93E-07
rs2975292	1419932	**rs1478435**	1454612	2.13E-07
rs13161905	1417212	rs6413429	1447027	2.46E-07
rs456774	1432202	rs403636	1438354	2.82E-07
rs7737692	1461167	rs2292023	1461389	5.14E-07
rs1048953	1438174	rs10063727	1455250	5.56E-07
rs1048953	1438174	rs72717506	1455799	5.56E-07
rs462053	1431554	rs403636	1438354	9.21E-07
rs28363174	1393038	rs460700	1429969	1.57E-06
rs638964	1433867	rs403636	1438354	1.75E-06
rs28363174	1393038	rs428280	1436476	1.87E-06
rs28363174	1393038	rs465130	1432876	2.02E-06
rs13161905	1417212	rs7737692	1461167	2.19E-06
rs28363174	1393038	rs465989	1432881	2.25E-06
rs28363174	1393038	rs458609	1434306	2.27E-06
rs28363174	1393038	rs393795	1428514	2.37E-06
rs638577	1433831	rs403636	1438354	3.26E-06
rs487781	1433931	rs403636	1438354	3.26E-06
rs11133778	1421543	rs11564751	1447223	3.61E-06
rs457702	1434430	rs403636	1438354	3.77E-06
rs460007	1431853	rs403636	1438354	4.14E-06
rs11133778	1421543	rs6413429	1447027	4.25E-06
rs456082	1430515	rs403636	1438354	4.80E-06

rs464061	1430244	rs403636	1438354	5.40E-06
rs410209	1430775	rs403636	1438354	5.40E-06
rs458860	1430933	rs403636	1438354	5.40E-06
rs57106193	1431123	rs403636	1438354	5.40E-06
rs463379	1431164	rs403636	1438354	5.40E-06
rs458632	1431302	rs403636	1438354	5.40E-06
rs460934	1431306	rs403636	1438354	5.40E-06
rs461677	1431992	rs403636	1438354	5.70E-06
rs459141	1432042	rs403636	1438354	5.70E-06
rs460000	1432825	rs403636	1438354	5.70E-06
rs250682	1427803	rs403636	1438354	6.22E-06
rs27048	1412645	rs393795	1428514	8.29E-06
rs409588	1430834	rs403636	1438354	8.52E-06

*Position, as per human genome version GRCh37.p13.
This table presents new data.

Supplementary Table 2. IDRs of *SLC6A3* TFs.

TF	Disorder Concensus*	MobiDB-lite Prediction*	Disordered Regions (residues)	TF Reference
GMEB1	5.15	17.23	360-388	PMID: 31175277
HEY1	60.44	51.65	154-186	PMID: 21290414
HIVEP2	36.55	2.9	1-93, 340-416, 543-563, 751-985, 1485-1603, 1882-1951, 2024-2129, 2242-2325, 2423-2446	PMID: 31586043
LMX1A	21.47	44.5	161-208, 252-285	PMID: 22564125
NFE2L1	9.93-63.49	14.94-15.75	281-344, 394-425	PMID: 30259411
NR4A2 (NURR1)	7.74	55.89	1-22, 334-357	PMID: 11238740
PITX3	68.66	65.67	1-71	PMID: 19780901
SP1	63.94	31.21	1-93, 109-141, 285-304, 329-395, 567-598	PMID: 15816870
SP3	14.72	14.72	1-53, 65-88, 301-338	PMID: 15816870
SRP54		80.47	353-416, 430-504	PMID: 30259411
MED1[#]	46.93	48.51	609-706, 792-820, 874-893, 947-1566	PMID: 33333019

* Scores are for human proteins based on MobiDB (PMID: 33237329):
Piovesan D, Necci M, Escobedo N, Monzon AM, Hatos A, Mičetić I, et al.
MobiDB: intrinsically disordered proteins in 2021.
Nucleic Acids Res . 2021 Jan 8;49(D1):D361-D367

[#] As a positive TF for RNA-related condensation mechanisms and also expressed in dopamine neurons.
This table presents new data.
